# Overexpression of *LINC00152* correlates with poor patient survival and knockdown impairs cell proliferation in lung cancer

**DOI:** 10.1038/s41598-017-03043-x

**Published:** 2017-06-07

**Authors:** Shumei Feng, Jie Zhang, Wenmei Su, Shengbin Bai, Lei Xiao, Xiuyuan Chen, Jules Lin, Rishindra M. Reddy, Andrew C. Chang, David G. Beer, Guoan Chen

**Affiliations:** 1 Xinjiang Medical University, Urumqi, China; 2 Section of Thoracic Surgery, University of Michigan, Ann Arbor, Michigan USA; 3 Xian Jiaotong University, Xi’an, China; 4 Guangdong Medical University, Zhanjiang, China; 5 Peking University People’s Hospital, Beijing, China

## Abstract

We employed RNA sequencing analysis to reveal dysregulated lncRNAs in lung cancer utilizing 461 lung adenocarcinomas and 156 normal lung tissues from 3 separate cohorts. We found that *LINC00152* was highly overexpressed in lung tumors as compared to their adjacent normal tissues. Patients with high *LINC00152* expression demonstrate a significantly poorer survival than those with low expression. We verified the diagnostic/prognostic potential of *LINC00152* expression in an independent cohort of lung tumor tissues using quantitative RT-PCR. After knockdown of *LINC00152* using siRNAs in lung cancer cell lines, both cell proliferation and colony formation were decreased. Cell fractionation and qRT-PCR analysis indicated that *LINC00152* is found mainly in the cytoplasm. Treatment with Trichostatin A in cell lines having low *LINC00152* expression indicated that histone acetylation may be one mechanism underlying *LINC00152* overexpression in NSCLC. Western blot analyses indicated that p38a, STAT1, STAT3, CREB1, CCNE1 and c-MYC proteins were decreased after *LINC00152* siRNA treatment. Our study indicates *LINC00152* plays an important role in lung tumor growth and is potentially a diagnostic/prognostic marker. Further characterization of *LINC00152* in regulating its target proteins may provide a novel therapeutic target of lung cancer.

## Introduction

It is estimated that 595,690 Americans will die from cancer in 2016, and more than one-quarter of these (158,080) will be due to lung cancer. Lung cancer continues to be the number one cause of cancer related death in both men and women worldwide^1^. While recent advances using screening CT scans are diagnosing early disease more frequently, the 5-year relative survival is still low (18%). These low survival rates are partly due to the fact that one-half of cases are diagnosed at a higher stage, for which 5-year survival is only 4%^1, 2^. While multiple molecular events converge to trigger unregulated growth, invasion, and metastasis in lung cancer, the exact mechanisms are not fully understood. Thus there is an urgent need for identification of markers that may aid in the early diagnosis or stratification of lung cancer as well as new therapeutic targets.

Accumulated evidence showed that more than 70% of the human genome is transcribed into primary RNA, but only about 2% encodes for peptide products, with the remainder being noncoding RNAs (ncRNAs)^3, 4^. These ncRNAs can be divided into two groups based on their transcript lengths: small ncRNAs, which are shorter than 200 bp, and long ncRNAs (lncRNAs), which are longer than 200 bp^5^. The lncRNAs are usually expressed in a tissue-specific pattern and show a low level of expression and demonstrate low sequence conservation as compared to protein coding RNAs. Through gene expression microarrays and RNA sequencing analysis, hundreds of lncRNAs have been reported to be dysregulated in lung cancer^6–8^; however, only a few have been well characterized regarding their functional role in cancer^9^. LncRNAs are thought to drive many important cancer phenotypes and disease-related pathways such as controlling cellular proliferation, invasion, development, lineage commitment, immune response, pluripotency and differentiation^10–13^. The cellular localization of lncRNAs may determine their function roles, e.g. nuclear lncRNAs are enriched for functions involving chromatin interaction, transcriptional regulation and RNA processing, while cytoplasmic lncRNAs can modulate mRNA stability or translation and influence cellular signaling cascades^14^.

*LINC00152* has been linked to several human cancers and promotes cell proliferation in gastric and hepatocellular carcinoma (HCC)^15, 16^. Additionally, it may also act as a potential prognostic biomarker and therapeutic target in colorectal cancer, clear cell renal carcinoma and HCC^17–19^. Moreover, since plasma levels of *LINC00152* are significantly elevated in patients with gastric cancer, this lncRNA has the potential to serve as a blood-based biomarker for this disease^20^. The expression of *LINC00152* and its functional roles in lung cancer, however, are unexplored. In this study, through analysis of RNA-Seq data from a large cohort of lung cancers, we demonstrate that *LINC00152* is highly expressed in lung cancer, and associated with poor patient survival. We validate its expression in an independent cohort of primary lung cancer using RT-PCR and explored its oncogenic functions in lung cancer cell lines, as well as the possible molecular mechanisms involved in lung cancer.

## Results

### Increased *LINC00152* expression is correlated with worse prognosis in patient with lung ADs

**Figure 1:**
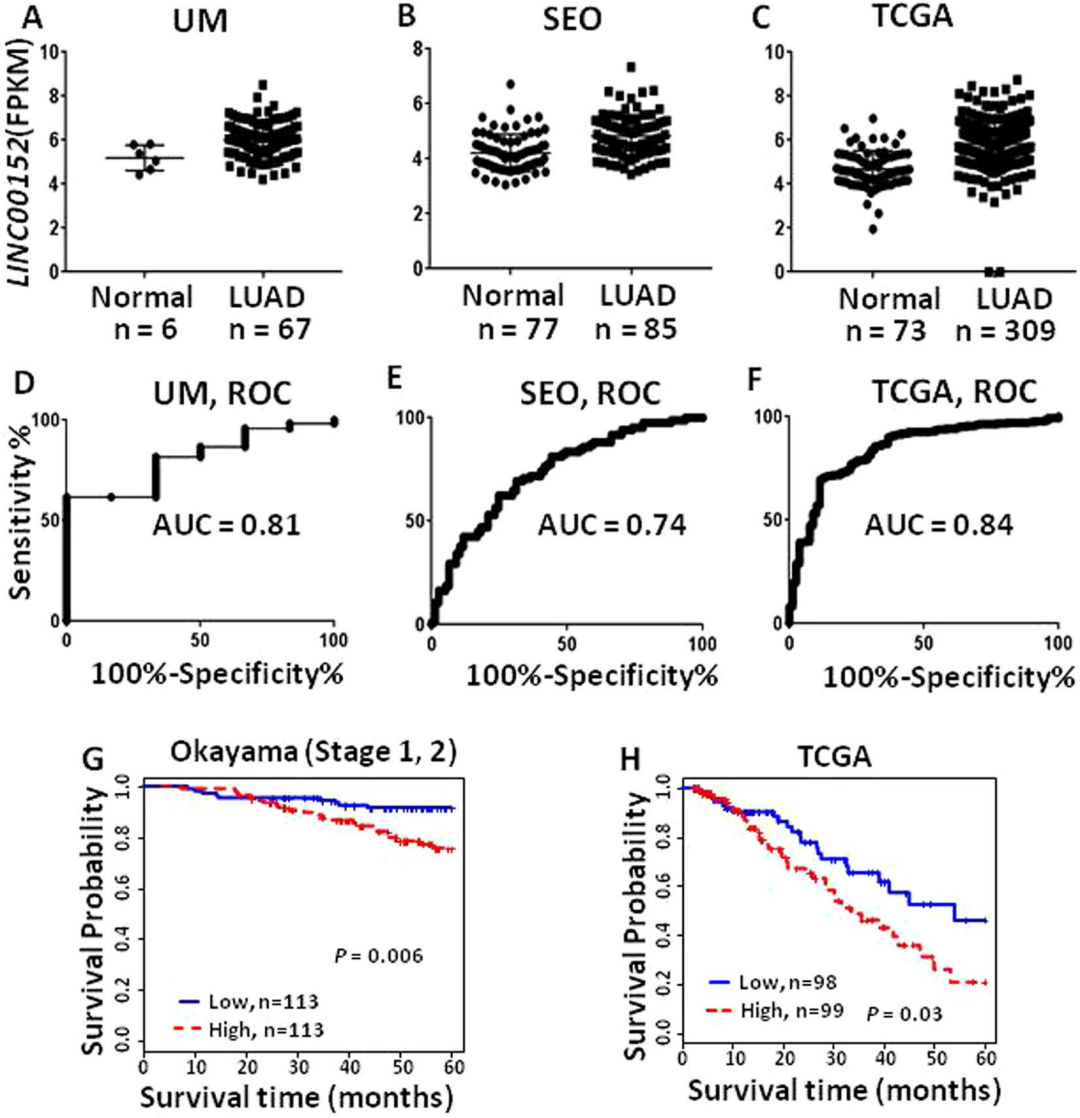
Differentially expression of *LINC00152* in lung ADs. (**A–C**) Scatterplots of *LINC00152* expression levels in lung AD and normal tissue samples in UM, Seo and TCGA RNA-Seq data sets (y-axis is log2 of FPKM value, LUAD vs. Normal, *p < *0.001 in all 3 data sets). (**D–F**) ROC curves with AUC values of *LINC00152* in UM (67 LUAD vs 6 N), Seo (85 LUAD vs 77 N) and TCGA (309 LUAD vs 73 N) RNA-Seq data sets. (**G** and **H**) Kaplan-Meier curves and log-rank test of *LINC00152* in the Okayama (226 LUADs), and TCGA (197 LUADs) data sets. Higher *LINC00152* expression was associated with poor patient survival.

To identify the dysregulated lncRNAs and their diagnostic potential in lung adenocarcinomas (LUAD), the largest subtype of NSCLC, we performed Receiver Operating Characteristic (ROC) curve analysis with combined RNA-Seq data from our cohort (UM 67 LUADs and 6 normal lung tissues), and two other independent published LUAD cohorts, Seo (85 LUADs and 85 normal lung tissues)^21^ and TCGA (309 LUADs and 73 normal lung tissues)^22^. We have identified lncRNAs differentially expressed in LUADs as compared to normal lung tissues^6^. Among the most highly overexpressed lncRNAs, *LINC00152* was found to be significantly increased in LUADs. Scatterplots showed that *LINC00152* maintained a high expression level in tumors (vs. normal) in the three cohorts (Fig. 1A–C). The area under the curve (AUC) values from ROC analysis were larger than 0.74 in all 3 cohorts (Fig. 1D–F) indicating that *LINC00152* may be potentially used as a novel diagnostic marker for this type of lung cancer. We next evaluated the association of *LINC00152* and patient survival in two independently published LUAD microarray data sets where survival information was available, Okayama *et al*. (226 LUADs, stage 1 and 2)^23^ and TCGA (197 LUADs, stage 1 to 3)^22^. Kaplan-Meier survival curves and log-rank tests showed that higher expression levels of *LINC00152* was significantly correlated with poor patient outcome in the Okayama (p = 0.001) and TCGA data sets (p = 0.03) (Fig. 1G,H), whereas patients with relatively lower levels of *LINC00152* expression showed better survival. Taken together, *LINC00152* was significantly overexpressed in LUAD across multiple studies and overexpression was a predictor of poor patient survival in LUADs.

### Validation of *LINC00152* expression pattern in an independent cohort of LUADs by qRT-PCR

**Figure 2:**
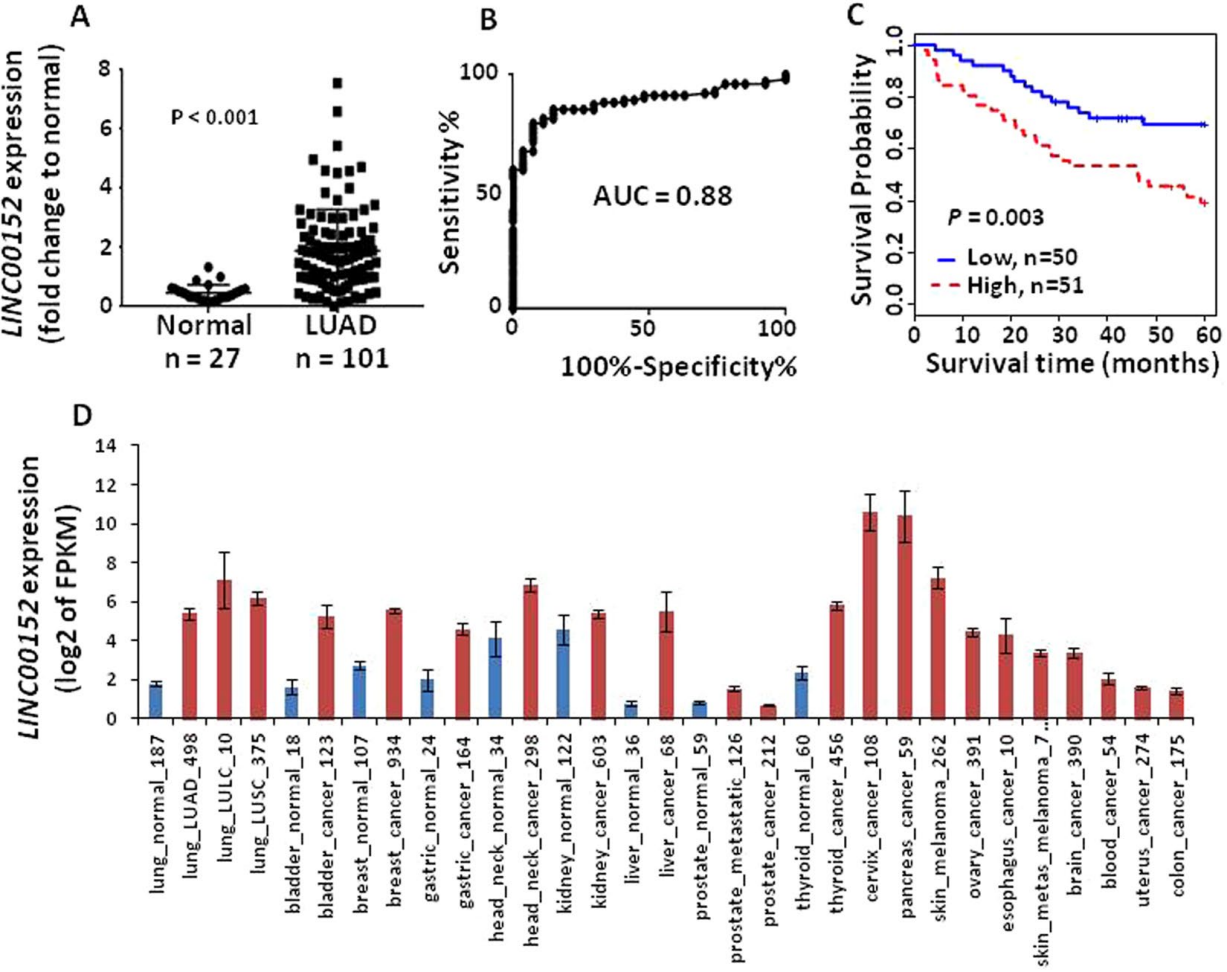
qRT-PCR validation of *LINC00152* expression in an independent data set (**A**–**C**) and *LINC00152* expression in other tumors types (**D**). (**A**) Scatterplot indicating *LINC00152* expression was higher in tumor (measured by RT-PCR, y-axis is fold change to mean of normal tissues, LUAD vs. normal, p < 0.001 by t test). (**B**) ROC curve indicated an excellent AUC (0.88) for classifying the 101 LUAD from 27 normal lung tissues based on *LINC00152* expression. (**C**) Kaplan-Meier curve indicated higher *LINC00152* expression was unfavorable for patient survival. (**D**) Comparison of *LINC00152* expression in cancers (red) and matched normal (blue) tissues (mean + SEM, log2 of FPKM from RNA-seq data). Lung, bladder, breast, gastric, head and neck, kidney, liver, and thyroid cancers were significantly higher in tumor (vs. normal, t test, p < 0.05). Case number of each cancer is indicated. The original data was downloaded from miTranscriptome with modification.

To verify the *LINC00152* expression pattern discovered from RNA-Seq and microarray data sets, we performed qRT-PCR using mRNA from an independent cohort from UM including 101 LUADs and 27 normal lung tissues. The results showed that *LINC00152* expression levels were significantly higher in LUAD as compared to normal lung tissues (p < 0.001) (Fig. 2A). The AUC was 0.88 indicated *LINC00152* expression levels could classify the tumors from normal lung (Fig. 2B). Kaplan-Meier survival curve and the log-rank test indicated that higher expression of *LINC00152* was significantly related to worse patient survival (p = 0.003) (Fig. 2C). We also analyzed *LINC00152* expression levels and other clinical variables from this validation set, however, no evidence was obtained to support the association between *LINC00152* expression and age, gender, smoking, differentiation, tumor stage, lymph node or *KRAS* mutation status (Supplementary Table 1).

Overexpression of *LINC00152* was reported in colorectal cancer, clear cell renal carcinoma and HCC; however to determine if *LINC00152* expression was higher in other cancers, we analyzed RNA-Seq expression data including 6,220 cancers from the MiTranscriptome database^24^. We found that *LINC00152* was increased in most cancers including bladder, breast, gastric, head/neck, kidney, liver, thyroid, cervix, blood, uterus, and colon cancers (FRKM log2 value > 1.4) but not prostate cancer (FRKM log2 = 0.67) (Fig. 2D). Interestingly, we found that squamous cell (LUSC) and large cell (LULC) lung carcinomas also highly expressed *LINC00152* (vs. normal). These results indicated that *LINC00152* was highly expressed not only in lung cancer but also in other type of cancers and could be potentially useful as a general cancer marker.

### *LINC00152* overexpression caused by histone acetylation

**Figure 3:**
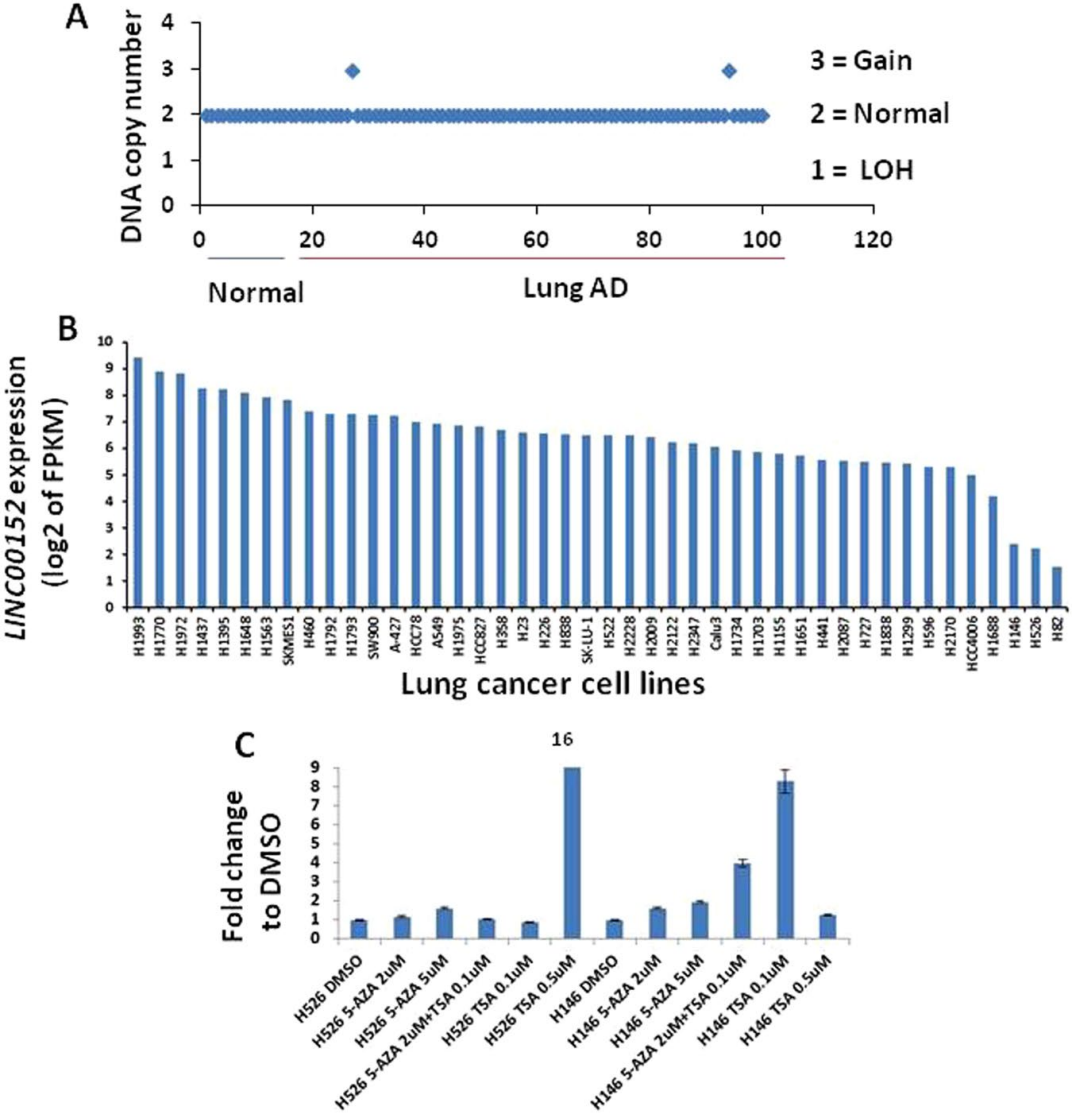
(**A**) No amplification of the *LINC00152* region was detected as determined by Affymetrix SNP6.0 analysis of 90 lung adenocarcinomas and 10 normal lung tissues. Two tumors with gains were found. (**B**) *LINC00152* maintains a high expression level in most of the non-small cell lung cancer cell lines according to the RNA-Seq data (log2 of FPKM value, p < 0.05), whereas, the three small cell lung cancer cell lines, H526, H146 and H82, have a relative low expression of *LINC00152*. (**C**) 5-aza-2-deoxycytidine 5-aza and Trichostatin A (TSA) treatment indicates that deacetylation might result in the low expression of *LINC00152* in H146 and H526 cells.

Gene expression could be influenced by genomic copy number changes, or specific transcription factors coupled with changes in histone and DNA modifications in its gene promoter. We examined DNA copy number changes of the *LINC00152* genomic locus by Affymetrix SNP 6.0 arrays on 90 lung adenocarcinomas and 10 normal lung tissues (unpublished data) and did not find any apparent amplification except for small gains in two cases (Fig. 3A). To test whether *LINC00152* expression is related to histone acetylation and promoter DNA methylation, we first analyzed its expression levels in 33 lung cancer cell lines from RNA-Seq data. We found that *LINC00152* was highly expressed in 30 NSCLC cell lines with lower expression in 3 small cell lung cancer cell lines, H146, H526 and H82 (Fig. 3B). We hypothesized that histone deacetylation or promoter DNA methylation may be the mechanisms causing the low expression of *LINC00152* in H146, H526 and H82 cells. We then treated these cell lines with 5-aza-2-deoxycytidine (5-AZA) and/or Trichostatin A (TSA) and found that *LINC00152* expression was increased by 16-fold in H526 and 8-fold in H146 cells after TSA treatment, whereas, 5-AZA didn’t change *LINC00152* expression (Fig. 3C), indicating that histone acetylation could be one mechanism causing *LINC00152* overexpression in NSCLC. We don’t know the reason so far why H146 cells with higher concentrations of TSA (0.5uM) lost the increase in expression of LINC00152. But, we found that these two small cell lung cancers have a different cell growth reaction upon *LINC00152* siRNA treatment (We described the results in Supplementary Figure S3).

### *LINC00152* knockdown decreased cell proliferation and colony formation in lung cancer cells

**Figure 4:**
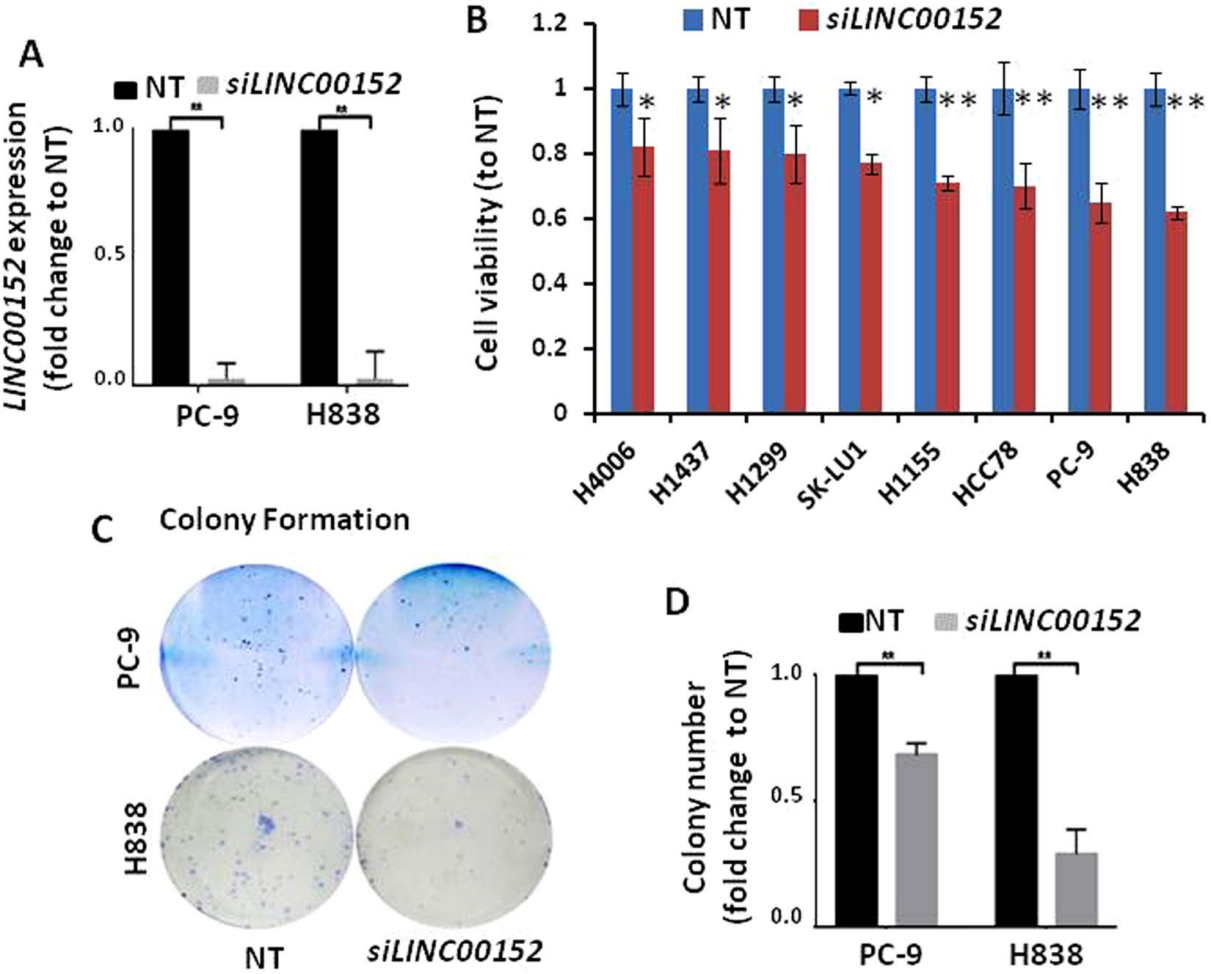
Effects of *LINC00152* on cell viability. (**A**) *LINC00152* expression was decreased more than 90% after *LINC00152* knockdown with siRNA on PC-9 and H838 cells measured by RT-PCR. (**B**) Cell proliferation measured by WST-1 assays was conducted on 8 lung cancer cell lines to determine the effect on cell viability after *LINC00152* siRNA transfection for 96 hours. Values indicate the mean + SD from three independent experiments. **p < 0.01, *p < 0.05. (**C** and **D**) Colony formation was decreased after *LINC00152* siRNA transfection on PC-9 and H838 cells. **p < 0.01.

Based on RNA-Seq value in Fig. 3B, all lung cancer cell lines have higher *LINC00152* expression level. In order to confirm the expression, we performed RT-PCR for LINC00152 expression on the cells which will be used for proliferation assay. A significant correlation between RNA-Seq and RT-PCR was found using Pearson correlation analysis (Supplementary Figure S1). Small cell lung cancer cell line H526 still is the lowest expression for LINC00152. To minimize the possibility of off-target effects, we used SMARTpool gene specific siRNAs whose knockdown efficiency was greater than 80–90% as determined by qRT-PCR (Fig. 4A and Supplementary Figure S2). To explore the oncogenic function of *LINC00152*, we examined cell proliferation status after *LINC00152* knockdown by siRNA in 12 lung cancer cell lines. A significant decrease in cell proliferation rate (from 18% to 38%) was identified in 9 out of 12 cells measured by WST-1 (Fig. 4B and Supplementary Figure S3A). The cell growth of three cell lines, H1975, H1650 and H146, was not affected by *LINC00152* knockdown. PC-9 (EGFR mutant and EGFR tyrosine kinase inhibitor (TKI) sensitive cell line) and H838 (EGFR wild type cell line) cells were the most significantly affected (decreased by 36% and 38%, respectively) and were then chosen for colony formation assay. The number of colonies was markedly reduced after LINC00152 siRNA knockdown in PC-9 and H838 cells (Fig. 4C and D). LINC00152 did not affect cell invasion using Boyden chamber matrix assays in these two lung cancer cell lines (data not shown).

We didn’t find a significant correlation between the *LINC00152* expression and the inhibition rate of cell growth by *LINC00152* knockdown (Supplementary Figure S3B). The cell growth in H1975 and H1650 cells were not affected by *LINC00152* siRNA knockdown although these cells have relative higher level of *LINC00152* expression. Whereas, the cell growth in H526 was affected although this cell has relative low level of *LINC00152* expression. But the cell growth in H146, another low level of *LINC00152* expression cell line, was not affected. This indicates that cell growth will be affected if *LINC00152* expression reaches a certain level and the genomic background may be the key factor regarding the role of *LINC00152* in cell growth.

### Proteins/mRNAs regulated by *LINC00152* in lung cancer cells

**Figure 5:**
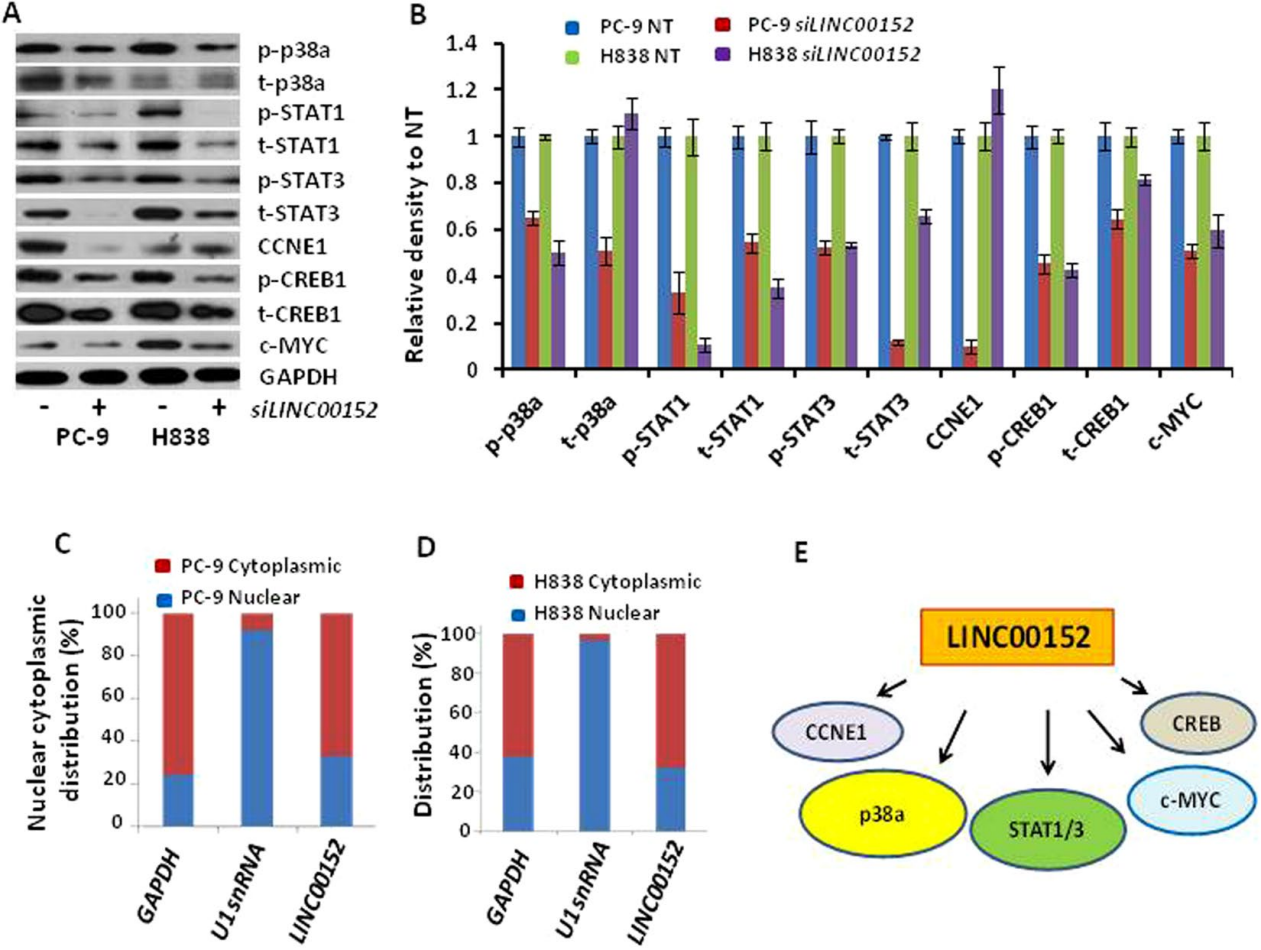
Proteins regulated by *LINC00152* and cellular location of *LINC00152*. (**A**) Western blot showing the altered protein expression of p38α, STAT1, STAT3, CREB1, CCNE1, and c-MYC after *LINC00152* knockdown with siRNA. (**B**) Quantitative analysis of image in A using ImageJ software. GAPDH was used as a protein loading control. Each individual band was divided by GAPDH first, then divided by NT. So each protein value (siRNA treated) was relative to their NT (NT = 1). (**C** and **D**) qRT-PCR showing the nuclear and cytoplasmic fractions of *LINC00152* in PC-9 and H838 cells. GAPDH was used as cytoplasmic control and U1 snRNA as nuclear control. *LINC00152* is primarily in cytoplasmic (67.7%). (**E**) Summary of proteins affected by *LINC00152* in this study. As indicated in Fig. 5A and B, all proteins were decreased after *LINC00152* knockdown, but CCNE1 and total p38a proteins were not changed in H838 cells.

To provide molecular mechanistic insight into *LINC00152* role in regulating lung cancer cell proliferation, we first performed receptor tyrosine kinase (RTK) phosphorylation antibody array analysis, which includes 49 different phosphorylated proteins covering most of the cancer-related pathways. The hosphorylation levels of STAT1 and STAT3 proteins were found to be decreased when *LINC00152* was knocked down by siRNA at 72 hours (Supplementary Fig. S4A and B). In order to confirm and identify more altered proteins regulated by *LINC00152*, we performed Western blot on several cell growth-related proteins. As indicated in Fig. 5A and B, p38α, STAT1, STAT3, CCNE1, CREB1 and c-MYC proteins were decreased after *LINC00152* siRNA treatment in PC-9 and H838 cell lines. The total proteins of p38α and CCNE1 in H838 cell line were not changed indicating a different regulation mechanism for p38α and CCNE1 by *LINC00152* between PC-9 and H838 cells. We also performed the mRNA expression of these genes (Supplementary Fig. S5), we found that STAT3 mRNA was decreased by 30–40% in both cells, whereas CCNE1 mRNA in H838 was increased by 1.5 fold. Other gene mRNAs (p38a, STAT1, CREB1 and c-MYC) were not changed. These results indicated that *LINC00152* regulates STAT3 may be through transcription regulation, whereas other proteins, such as p38a, STAT1, CREB1, and c-MYC may be regulated at the post-transcription. The mechanism of these proteins regulated by *LINC00152*, such as if through lysosome or ubiquitin dependent process, is warranted to be analyzed in the future.

Since EGFR signaling was reported to be involved in *LINC00152* promoting cell proliferation in gastric cancer^15^, we performed Western blot on EGFR, AKT and ERK1/2 proteins. We found that these proteins were not changed after *LINC00152* siRNA treatment at 72 hours (Supplementary Fig. S4C) indicating that EGFR signaling was not involved in *LINC00152* regulation in these lung cancer cells.

In order to evaluate the relationship between p38α and STAT3, we performed p38α and STAT3 siRNA knockdown on PC-9 and H838 cells. As shown in Supplementary Fig. S6, the ratios of p-STAT3/t-STAT3P were increased by 1.6–1.7 fold (vs. NT) in both cells after p38a knockdown and the mRNA levels were not changed indicating that p38α may regulate STAT3 at the protein level. Figure 5E summarized the proteins regulated by *LINC00152* uncovered in this study. More experiments such as STAT1, CCNE1, CREB1 and c-MYC expression changing after p38a or STAT3 knockdown or overexpression is warranted.

Finally, we examined the cellular localization of *LINC00152*. RNAs were isolated from total, nuclear and cytoplasmic fractions of these two cell lines. Quantitative RT-PCR indicated that the expression of *LINC00152* was mainly located in the cytoplasm in both PC-9 (67.2%) and H838 cells (67.7%) (Fig. 5C and D). *LINC00152* expression primarily in cytoplasm may support that *LINC00152* plays roles at post-transcriptional level which is different from a recent report showing *LINC00152* interaction with EZH2 and transcriptional control of target genes in A549 and SPCA1 cell lines^25^.

## Discussion

As highly tissue-specific drivers of cancer phenotypes, lncRNAs are potentially prime targets for cancer therapy. Many studies have confirmed their utility as biomarkers not only in cancer diagnosis but also for patient prognosis across a variety of cancers^26–30^.

*LINC00152* has been reported to be highly expressed in hepatocellular carcinoma, gastric cancer and clear cell renal carcinoma and is involved in the cancer progression^15–19, 25^. In the present study, we found that the average levels of *LINC00152* in LUAD tissues was significantly higher than those in corresponding normal tissues, and higher expression of *LINC00152* was associated with a poor patient survival. These results suggest that *LINC00152* may be potentially useful as a marker for lung cancer diagnosis and an indicator of poor survival. We found that knockdown of *LINC00152* suppressed tumor cell proliferation and colony formation capability of lung cancer cells but did not affect tumor cell invasion, which was consistent with the results observed in both gastric cancer and hepatocellular carcinomas^15, 16^.

In different cancer types, and even in different cancer cells, lincRNAs may regulate oncogenesis by different molecular mechanisms. In one study, *LINC00152* was found to promote tumor growth through an EGFR-mediated PI3/AKT pathway in gastric cancer cell lines, MGC803 and HGC-27^15^, whereas in another study with gastric cell lines BGC-823 and SGC-7901 cells, *LINC00152* promoted GC tumor cell cycle progression by binding to enhancer of zeste homolog 2 (EZH2), thus silencing the expression of p15 and p21^31^. In hepatocellular carcinoma, *LINC00152* appears to activate the rapamycin (mTOR) pathway by binding to the promoter of EpCAM through cis-regulation, which was confirmed by the Gal4-λN/BoxB reporter system^16^. In the present study in lung cancer, *LINC00152* knockdown leads to reduced several cell growth-related proteins such as STAT3, p38α, CREB1, STAT1, c-MYC and CCNE1. This suggests that the molecular signaling affected by *LINC00152* in lung cancer may be different from gastric and liver cancers.

The p38 MAPK pathway is known to regulate gene expression, allowing cells to respond to various extracellular stresses, and different stimuli can consequently activate p38 MAPKs and influence a variety of downstream molecules. CREB1, STAT1 and STAT3 are key factors for p38α signaling^32^. CREB1 influences cell cycle arrest through increasing expression of CCNE1. The p38 MAPK-regulated-CREB1 pathway was shown to contribute to selenite-induced colorectal cancer cell apoptosis *in vitro* and *in vivo* ^33^. Pretreatment with the p38 inhibitor SB203580, increased expression of p-STAT3 in the lung adenocarcinoma cell line A549^34^ indicating that STAT3 was downstream of the ERK and p38 signaling pathways. We found that the ratios of p-STAT3/t-STAT3P were increased by 1.6–1.7 fold (vs. NT) in both PC-9 and H838 cells after p38a knockdown and the mRNA levels were not changed indicating that p38α may regulate STAT3 at the protein level.

STAT3 was shown to directly or indirectly upregulate the expression of genes required for uncontrolled proliferation and survival, including the genes that encode c-MYC, cyclin D1 and cyclin D2, BCL-XL, MCL1 and survivin^35, 36^. The increased expression of STAT1 and STAT3 may also positively affect c-Myc, thus precipitating a series of events during oncogenesis including apoptosis inhibition, cell proliferation, angiogenesis and anti-immune responses. We found that STAT3 mRNA and protein were decreased after *LINC00152* knockdown indicated that *LINC00152* regulated STAT3 may be at the transcription level. Based on our finding that *LINC00152* was mainly located in the cytoplasmic of lung cancer cells, the regulation process targeted to p38α, STAT1, CREB1 and c-MYC growth related proteins may be at the post-transcriptional level such as protein degradation mechanism. The detailed mechanism of these proteins regulated by *LINC00152*, such as if through lysosome or ubiquitin dependent process, is warranted to be further studied.

Taken together, our study demonstrated that *LINC00152* was up-regulated in human LUAD, and was correlated with the poor survival of LUAD patients. Its increased expression of *LINC00152* might be involved in lung cancer development, and *LINC00152* may serve as a potential marker for diagnosis and prognosis. Further characterization of *LINC00152* in regulating STAT3, p38a and other proteins may provide a novel therapeutic target of lung cancer.

## Materials and Methods

### Cell culture

Human lung cancer cell lines were purchased from the American Type Culture Collection (ATCC, Manassas, VA). All cell lines were routinely maintained in RPMI 1640 supplemented with 10% FBS. All cell lines were cultured in a humidified incubator in 5% CO_2_ atmosphere at 37 °C. All cell lines were genotyped for identity at the University of Michigan Sequencing Core and were tested routinely for mycoplasma contamination.

### Lung tissue samples

Lung cancer tissues were collected from patients undergoing curative cancer surgery from 1991 to 2013 at the University of Michigan Health System. Informed consent was obtained from all patients with experimental protocols receiving approval from the University of Michigan Institutional Review Board and Ethics Committee. The methods were carried out in accordance with approved guidelines. The specimens collected from surgery were freshly frozen in liquid nitrogen and then stored at −80 °C. Frozen tissues for regions containing a minimum of 70% tumor cellularity, defined by cryostat sectioning, were utilized for RNA isolation. All included patients did not receive preoperative radiation or chemotherapy.

### 5-aza-2-deoxycytidine and Trichotatin A Treatment

Two cell lines, H526 and H146, showing low expression of *LINC00152*, were treated with 5-aza-2-deoxycytidine (5-AZA) or Trichotatin A (TSA). H526 and H146 were seeded in 6-well plates at the desired density. After treatment with 5-AZA (2 µM and 5 µM) or TSA (0.1 µM and 1 µM) for 48 hours, RNA from each treatment were collected for *LINC00152* expression analysis by qRT-PCR.

### siRNA transfection

Cells were seeded in 6-well or 96-well plates at the desired density overnight. The target siRNA oligonucleotides or NT (non-targeting RNA) controls with a final concentration of 10 nM were transfected with lipofectamine RNAiMax Reagent (Invitrogen, USA) in OptiMEM medium according to the manufacturer’s instructions. siRNA knockdown efficiency was confirmed by qRT-PCR.

### Cell proliferation and colony formation

All the cell lines were plated in 96-well plates at the desired density. After plating for 24 hours, *LINC00152* siRNA and NT control were transfected with Lipofectamine RNAiMax Reagent in OptiMEM medium. After transfection with 10 nM LINC00152 siRNA for 96 hours, cell proliferation reagents (WST-1) (Roche) were added to each well, and the absorbance was measured at wavelengths of 450 nm and 630 nm, according to the manufacturer’s instructions. The cell viability percentages were calculated by normalizing to control siRNA.

Colony formation assay was performed to measure the number of viable cells after *LINC00152* knockdown by siRNA. Forty-eight hours after siRNA transfection, PC-9 and H838 cells were trypsinized and counted, and an equal amount of cells (100 or 500 cells/well) were then seeded evenly onto 6-well plates in triplicate. Ten to fourteen days later, colonies were stained with 0.5% crystal violet, and colonies with more than 50 cells were counted.

### RNA subcellular isolation and qRT-PCR

To identify the subcellular localization of *LINC00152*, all the cell lines were plated in 100 mm plates. The cells were harvested at 80–90% confluence. The cytoplasmic and nuclear RNA were extracted following the instructions for the RNA Subcellular Isolation Kit (Active Motif). Adding complete lysis buffer to each cell pellet and centrifuging each sample, cytoplasmic RNA was harvested from the supernatant, while nuclear RNA was present in the pellet. The cDNA was synthesized with the High Capacity cDNA Reverse Transcription kit (Applied Biosystems). Quantitative RT-PCR was performed using Power SYBR Green master Mix (Thermo Fisher Scientific, USA) and performed with an ABI StepOne Real-Time PCR System (Applied Biosystems). Each sample was analyzed in duplicate. The housekeeping gene GAPDH was used as loading controls.

### Microarray and RNA sequencing datasets

One published Affymetrix microarray data set representing 226 primary lung AD tissues^23^ was used for survival analysis. The CEL files of microarray data were normalized using the Robust Multi-array Average (RMA) method^37^. We also obtained RNA-Seq data sets from Seo^21^ and TCGA^22^ consisting of a total of 394 ADs, 212 SCCs and 150 normal lung tissues. Expression levels of transcripts were represented as FPKM^38^. Our primary outcome was overall survival, censored at five years. The information concerning adjuvant chemotherapy or radiation therapy was obtained from the original papers.

### Receptor tyrosine kinases (RTK) signaling antibody array and Western blot analysis

To explore the possible regulation mechanisms of *LINC00152*, PC-9 and H838 cell lines were seeded on 6-well plates separately at a density of 10,000 cells per well. The cell lysates were collected after *LINC00152* siRNA treatment at 72 h. The pathscan RTK antibody array (Cell Signaling) was performed according to the manufacturer’s instructions. Western blots were also performed with 10 µg protein using polyacrylamide gel electrophoresis and transfer to nitrocellulose membranes. After being blocked for 1 h with 5% non-fat milk, the membranes were incubated with primary antibodies on a rolling shaker overnight at 4 °C. After incubation with a secondary antibody for 1 hour at room temperature, the membranes were developed using the ECL kit (Amersham, Arlington Heights, IL) and exposed to X-ray film.

### Statistical analysis

Data were analyzed using GraphPad Prism 6 (GraphPad software) and R software. To evaluate the diagnostic potential of *LINC00152* in LUAD vs. normal, Receiver Operating Characteristic (ROC) curve analysis was used. It showed the tradeoff between sensitivity and specificity (any increase in sensitivity will be accompanied by a decrease in specificity) for the different possible cut-points of a diagnostic test. The diagnostic accuracy was measured by the AUC (area under the curve). An AUC of 1 represented a perfect test; an AUC of 0.5 represented an imprecise test. The data are presented as the mean ± SEM from triplicate experiments and additional replicates as indicated. The significant differences between groups were calculated with Student’s t-test or paired t-test. Survival analysis was performed using the Kaplan–Meier method, and the curves were compared using the log-rank test. A p value < 0.05 was considered statistically significant.

## Electronic supplementary material

Supplementary data

## References

[1] Siegel RL, Miller KD, Jemal A (2016). Cancer statistics, 2016. CA Cancer J Clin.

[2] Torre LA, Siegel RL, Jemal A (2016). Lung Cancer Statistics. Adv Exp Med Biol.

[3] Haemmerle, M. & Gutschner, T. Long non-coding RNAs in cancer and development: where do we go from here? *Int J Mol Sci* **16**, 1395–1405, doi:ijms16011395 (2015). 10.3390/ijms16011395 PMC4307309 25580533

[4] Li, C. H. & Chen, Y. Targeting long non-coding RNAs in cancers: progress and prospects. *Int J Biochem Cell Biol* **45**, 1895–1910, doi:S1357-2725(13)00174-X (2013). 10.1016/j.biocel.2013.05.030 23748105

[5] Ma, L., Bajic, V. B. & Zhang, Z. On the classification of long non-coding RNAs. *RNA Biol* **10**, 925–933, doi:24604 (2013). 10.4161/rna.24604 PMC4111732 23696037

[6] Wang, L. *et al*. Non-coding RNA LINC00857 is predictive of poor patient survival and promotes tumor progression via cell cycle regulation in lung cancer. *Oncotarget* **7**, 11487–11499, doi:7203 (2016). 10.18632/oncotarget.7203 PMC4905488 26862852

[7] White NM (2014). Transcriptome sequencing reveals altered long intergenic non-coding RNAs in lung cancer. Genome Biol.

[8] Xu G (2014). Long noncoding RNA expression profiles of lung adenocarcinoma ascertained by microarray analysis. PLoS One.

[9] Sang H, Liu H, Xiong P, Zhu M (2015). Long non-coding RNA functions in lung cancer. Tumour Biol.

[10] Yuan SX (2012). Long noncoding RNA associated with microvascular invasion in hepatocellular carcinoma promotes angiogenesis and serves as a predictor for hepatocellular carcinoma patients’ poor recurrence-free survival after hepatectomy. Hepatology.

[11] Hu, X. *et al*. A functional genomic approach identifies FAL1 as an oncogenic long noncoding RNA that associates with BMI1 and represses p21 expression in cancer. *Cancer Cell* **26**, 344–357, doi:S1535-6108(14)00300-6 (2014). 10.1016/j.ccr.2014.07.009 PMC4159613 25203321

[12] Gupta, R. A. *et al*. Long non-coding RNA HOTAIR reprograms chromatin state to promote cancer metastasis. *Nature* **464**, 1071–1076, doi:nature08975 (2010). 10.1038/nature08975 PMC3049919 20393566

[13] Schmitt AM (2016). An inducible long noncoding RNA amplifies DNA damage signaling. Nat Genet.

[14] Batista PJ, Chang HY (2013). Long noncoding RNAs: cellular address codes in development and disease. Cell.

[15] Zhou J (2015). Linc00152 promotes proliferation in gastric cancer through the EGFR-dependent pathway. J Exp Clin Cancer Res.

[16] Ji, J. *et al*. LINC00152 promotes proliferation in hepatocellular carcinoma by targeting EpCAM via the mTOR signaling pathway. *Oncotarget* **6**, 42813–42824, doi:5970 (2015). 10.18632/oncotarget.5970 PMC4767473 26540343

[17] Qiu, J. J. & Yan, J. B. Long non-coding RNA LINC01296 is a potential prognostic biomarker in patients with colorectal cancer. *Tumour Biol* **36**, 7175–7183, doi:10.1007/s13277-015-3448-5 (2015). 10.1007/s13277-015-3448-5 25894381

[18] Wu Y (2016). Long non-coding RNA Linc00152 is a positive prognostic factor for and demonstrates malignant biological behavior in clear cell renal cell carcinoma. Am J Cancer Res.

[19] Li, J. *et al*. HULC and Linc00152 Act as Novel Biomarkers in Predicting Diagnosis of Hepatocellular Carcinoma. *Cell Physiol Biochem* **37**, 687–696, doi:000430387 (2015). 10.1159/000430387 26356260

[20] Li Q (2015). Plasma long noncoding RNA protected by exosomes as a potential stable biomarker for gastric cancer. Tumour Biol.

[21] Seo, J. S. *et al*. The transcriptional landscape and mutational profile of lung adenocarcinoma. *Genome Res* **22**, 2109–2119, doi:gr.145144.112 (2012). 10.1101/gr.145144.112 PMC3483540 22975805

[22] Comprehensive molecular profiling of lung adenocarcinoma. *Nature* **511**, 543–550, doi:nature13385 (2014). 10.1038/nature13385 PMC4231481 25079552

[23] Okayama, H. *et al*. Identification of genes upregulated in ALK-positive and EGFR/KRAS/ALK-negative lung adenocarcinomas. *Cancer Res* **72**, 100–111, doi:0008-5472.CAN-11-1403 (2012). 10.1158/0008-5472.CAN-11-1403 22080568

[24] Iyer, M. K. *et al*. The landscape of long noncoding RNAs in the human transcriptome. *Nat Genet* **47**, 199–208, doi:ng.3192 (2015). 10.1038/ng.3192 PMC4417758 25599403

[25] Chen, Q. N. *et al*. Long intergenic non-coding RNA 00152 promotes lung adenocarcinoma proliferation via interacting with EZH2 and repressing IL24 expression. *Mol Cancer* **16**, 17, doi:10.1186/s12943-017-0581-3 (2017). 10.1186/s12943-017-0581-3 PMC5251237 28109288

[26] Yang, X. J., Huang, C. Q., Peng, C. W., Hou, J. X. & Liu, J. Y. Long noncoding RNA HULC promotes colorectal carcinoma progression through epigenetically repressing NKD2 expression. *Gene* **592**, 172–178, doi:S0378-1119(16)30619-9 (2016). 10.1016/j.gene.2016.08.002 27496341

[27] Guo, W. *et al*. Transcriptome sequencing uncovers a three-long noncoding RNA signature in predicting breast cancer survival. *Sci Rep* **6**, 27931, doi:srep27931 (2016). 10.1038/srep27931 PMC4919625 27338266

[28] Hong, H. H. **et al**. Long non-coding RNA UCA1 is a predictive biomarker of cancer. *Oncotarget*, doi:10142 (2016). 10.18632/oncotarget.10142 PMC5190109 27329842

[29] Wang Y (2016). The Long Noncoding RNA MALAT-1 is A Novel Biomarker in Various Cancers: A Meta-analysis Based on the GEO Database and Literature. J Cancer.

[30] Zhang, S. *et al*. lncRNA Up-Regulated in Nonmuscle Invasive Bladder Cancer Facilitates Tumor Growth and Acts as a Negative Prognostic Factor of Recurrence. *J Urol* **196**, 1270–1278, doi:S0022-5347(16)30559-6 (2016). 10.1016/j.juro.2016.05.107 27267320

[31] Chen, W. M. *et al*. Long intergenic non-coding RNA 00152 promotes tumor cell cycle progression by binding to EZH2 and repressing p15 and p21 in gastric cancer. *Oncotarget* **7**, 9773–9787, doi:6949 (2016). 10.18632/oncotarget.6949 PMC4891083 26799422

[32] Cuadrado, A. & Nebreda, A. R. Mechanisms and functions of p38 MAPK signalling. *Biochem J* **429**, 403–417, doi:BJ20100323 (2010). 10.1042/BJ20100323 20626350

[33] Hui, K. *et al*. The p38 MAPK-regulated PKD1/CREB/Bcl-2 pathway contributes to selenite-induced colorectal cancer cell apoptosis *in vitro* and *in vivo*. *Cancer Lett* **354**, 189–199, doi:S0304-3835(14)00437-6 (2014). 10.1016/j.canlet.2014.08.009 25128071

[34] Xue, P. *et al*. A novel compound RY10-4 induces apoptosis and inhibits invasion via inhibiting STAT3 through ERK-, p38-dependent pathways in human lung adenocarcinoma A549 cells. *Chem Biol Interact* **209**, 25–34, doi:S0009-2797(13)00316-5 (2014). 10.1016/j.cbi.2013.11.014 24300195

[35] Yu H, Jove R (2004). The STATs of cancer–new molecular targets come of age. Nat Rev Cancer.

[36] Harada D, Takigawa N, Kiura K (2014). The Role of STAT3 in Non-Small Cell Lung Cancer. Cancers.

[37] Irizarry RA (2003). Exploration, normalization, and summaries of high density oligonucleotide array probe level data. Biostatistics.

[38] Dhanasekaran SM (2014). Transcriptome meta-analysis of lung cancer reveals recurrent aberrations in NRG1 and Hippo pathway genes. Nature communications.

