# Supplementary material for: Overexpression of LINC00152 correlates with poor patient survival and knockdown impairs cell proliferation in lung cancer
Source: Sci Rep. 2017 Jun 7;7:2982. doi: 10.1038/s41598-017-03043-x (PMC5462773; doi:10.1038/s41598-017-03043-x)
Supplement: Supplementary file 1 — Supplementary data [file 41598_2017_3043_MOESM1_ESM.pdf]

**Overexpression of *LINC00152* correlates with poor patient survival and knockdown impairs cell proliferation in lung cancer**

Shumei Feng[1,2], Jie Zhang[3], Wenmei Su[4], Shengbin Bai[1], Lei Xiao[1], Xiuyuan Chen[5], Jules Lin[2], Rishindra M. Reddy[2], Andrew C. Chang[2], David G. Beer[2], Guoan Chen[2]

[1]Xinjiang Medical University, Urumqi, China; [2]Section of Thoracic Surgery, University of Michigan, Ann Arbor, Michigan, USA; [3]Xian Jiaotong University, Xi'an, China; [4]Guangdong Medical University, Zhanjiang, China, [5]Peking University People's Hospital, Beijing, China

Supplementary data

**Supplementary Table 1** *LINC00152* expression and clinico-pathological variables in lung cancer

| Characteristics | No. of patients | LINC00152 expression (Mean ± SD) | P Value t test |
|---|---|---|---|
| Age(years) | | | |
| ≥60 | 73 | 2.01±1.53 | |
| <60 | 28 | 1.67±1.14 | 0.21 |
| Gender | | | |
| Male | 48 | 1.73±1.32 | |
| Female | 53 | 2.02±1.47 | 0.29 |
| Differentiation | | | |
| Well | 28 | 1.52±1.24 | |
| Moderate | 38 | 2.04±1.27 | 0.10 |
| Poor | 34 | 2.0±1.63 | 0.19 |
| Tumor Stage: | | | |
| 1 | 59 | 1.71±1.56 | |
| 2 | 16 | 2.05±1.31 | 0.39 |
| 3 | 26 | 2.18±1.01 | 0.10 |
| Lymph Node | | | |
| N0 | 70 | 1.79±1.47 | |
| N1 | 12 | 2.02±1.44 | 0.63 |
| N2 | 19 | 2.13±1.11 | 0.28 |
| Smoking: Pack-years | | | |
| >60 | 16 | 2.35±1.48 | |
| ≤60 | 85 | 1.79±1.38 | 0.18 |
| Kras Mutation | | | |
| NO | 47 | 1.58±1.07 | |
| YES | 41 | 2.11±1.69 | 0.09 |

## Supplementary Figure S1

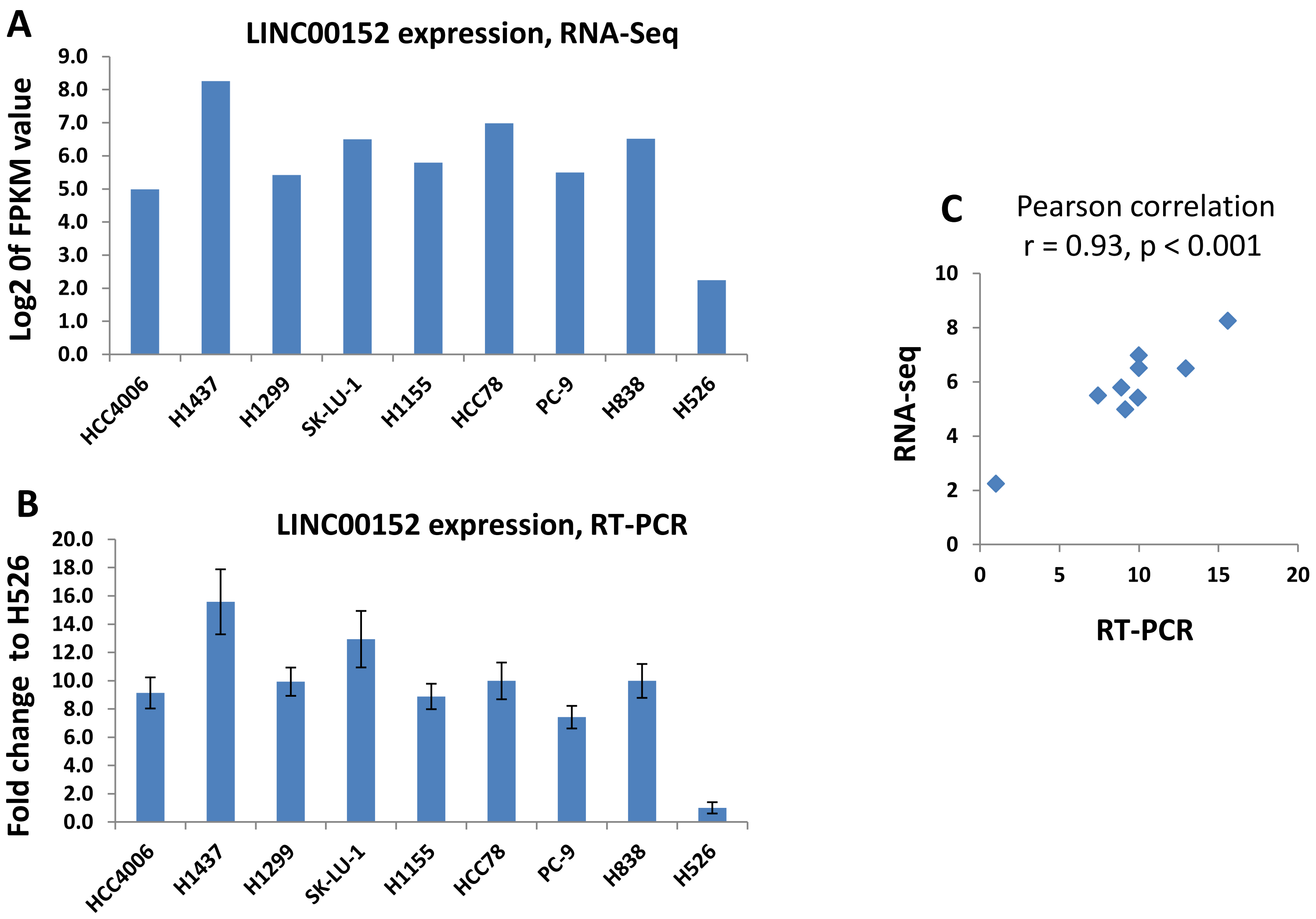


*LINC00152* expression in 9 lung cancer cell lines used in this study measured by RNA-Seq (**A**) and RT-qPCR (**B**). A significant correlation between these two methods was found using Pearson correlation analysis (**C**). Small cell lung cancer cell line H526 is the lowest expression for *LINC00152*. RNA-seq value of PC-9 was predicted vale based on RT-PCR value  as compared to other cells since we don't have the RNA-Seq data for PC-9 cell.

**Supplementary Figure S2**

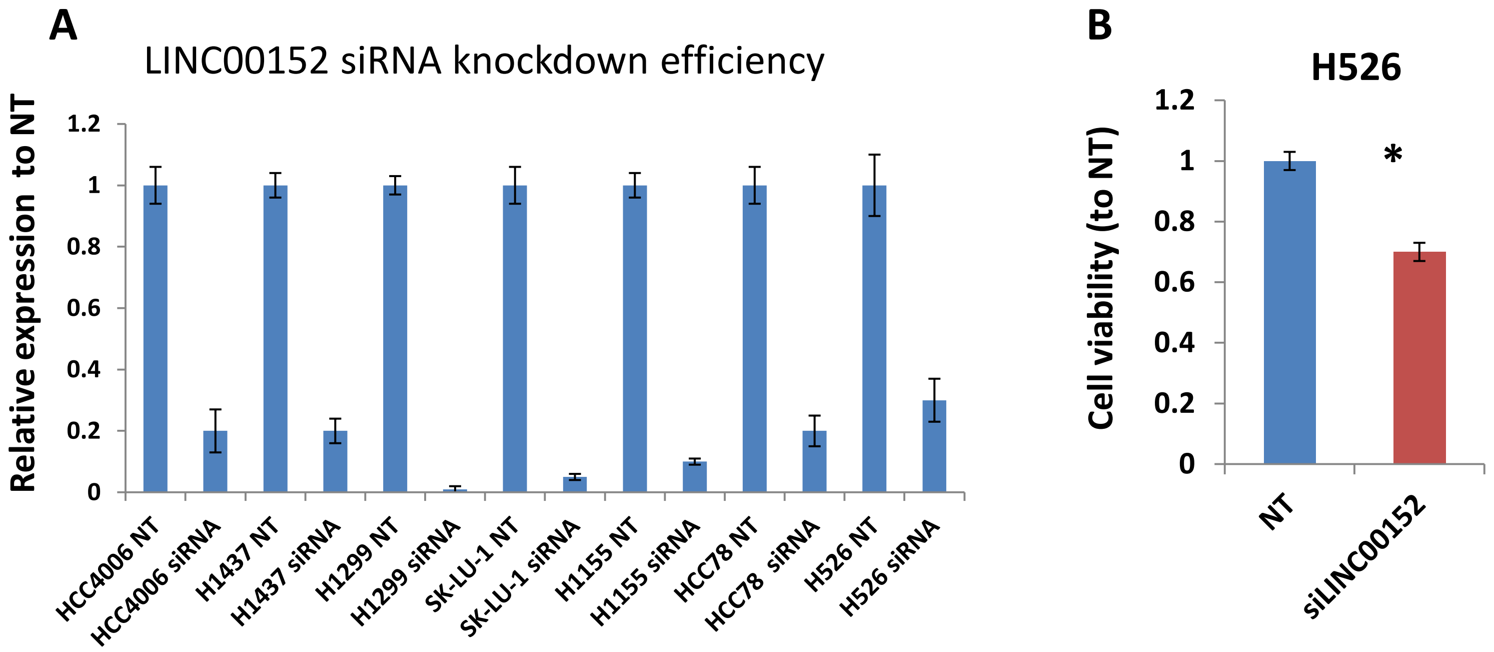


**A**, *LINC00152* siRNA (Dharmacon SMARTpool, 10nM) knockdown efficiency on lung cancer cell lines after siRNA treatment at 48 hours measured by RT-PCR. y-axis is relative *LINC00152* expression to NT after adjusted by GAPDH. **B**, Cell proliferation (measured by WST-1) of H526 cell line after *LINC00152* siRNA treatment at 96 hours.

## Supplementary Figure S3

**A**

| | RNA-seq | si*LINC00152* |
|---|---|---|
| Cell | LINC00152 | WST down |
| H838 | 6.5 | 38% |
| PC-9 | 5.5 | 35% |
| HCC78 | 7.0 | 30% |
| H526 | 2.2 | 30% |
| H1155 | 5.8 | 29% |
| SK-LU-1 | 6.5 | 23% |
| H1299 | 5.4 | 20% |
| H1437 | 8.3 | 19% |
| HCC4006 | 5.0 | 18% |
| H1975 | 6.9 | 0% |
| H1650 | 6.5 | 0% |
| H146 | 2.4 | 0% |

**B**

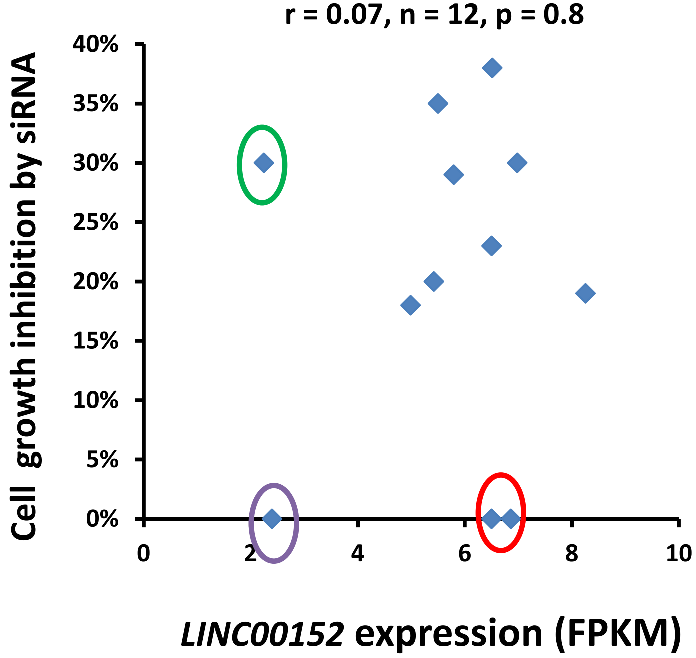


The correlation between *LINC00152* expression and rate of cell growth inhibition by *LINC00152* knockdown with siRNA. The cell growth in H1975 and H1650 cells (indicated by red cycle) were not affected by *LINC00152* siRNA knockdown although these cells have relative higher level of *LINC00152* expression. Whereas, cell growth in H526 (indicated by green cycle) was affected by *LINC00152* knockdown although this cell has relative low level of *LINC00152* expression. The cell growth in H146, another low level of *LINC00152* expression cell line, was not affected(indicated by purple cycle).

## Supplementary Figure S4

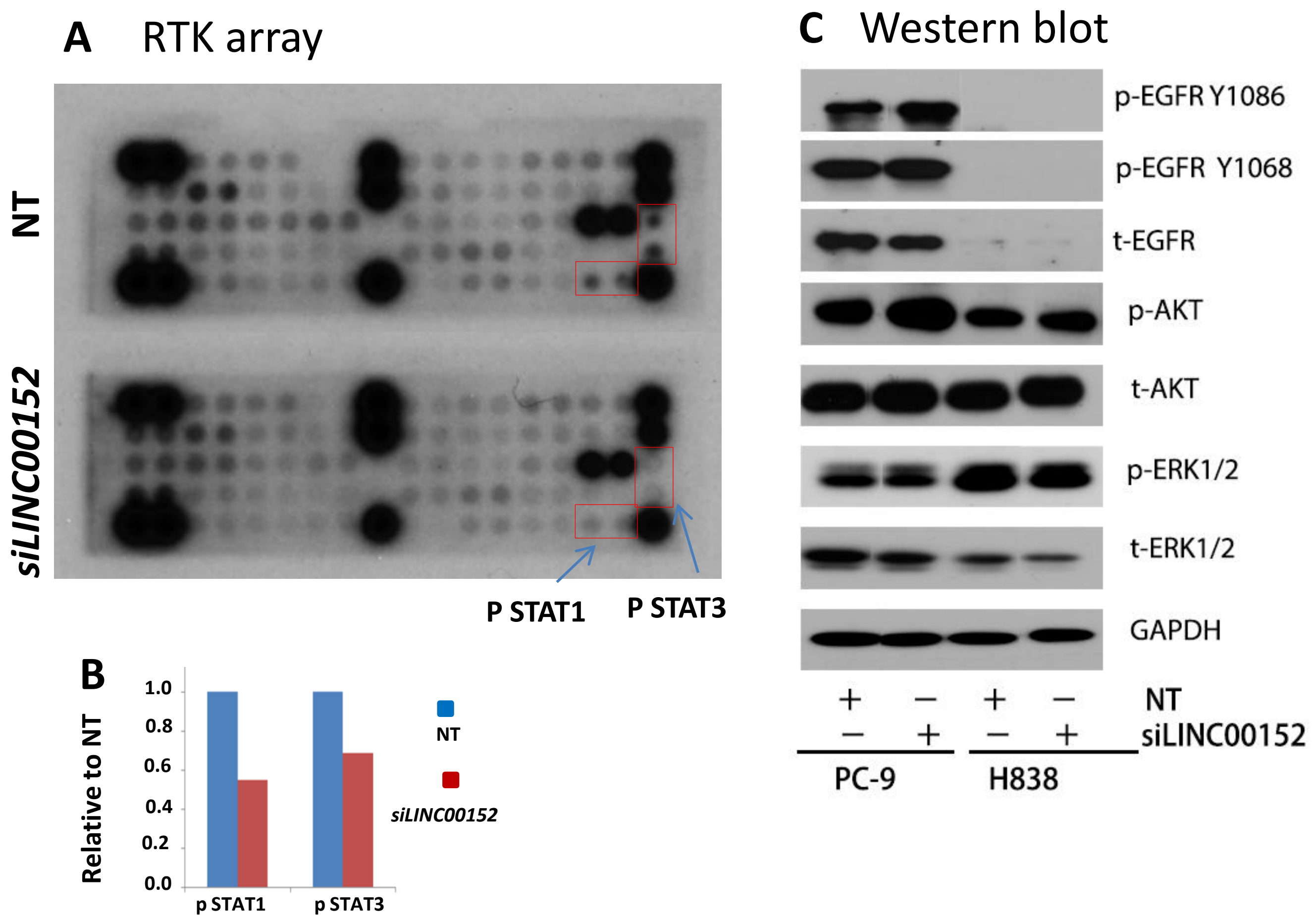


**A**, The whole blot of Receptor tyrosine kinases (RTK) array. Red box indicated that p STAT1 and p STAT3 expression were decreased. **B**, Quantitative analysis of p STAT1 and p STAT3 expression by Image J, relative to NT (NT=1). **C**, Western blot showing EGFR, AKT and ERK1/2 proteins were not changed after *LINC00152* siRNA treatment at 72 hours.

## Supplementary Figure S5

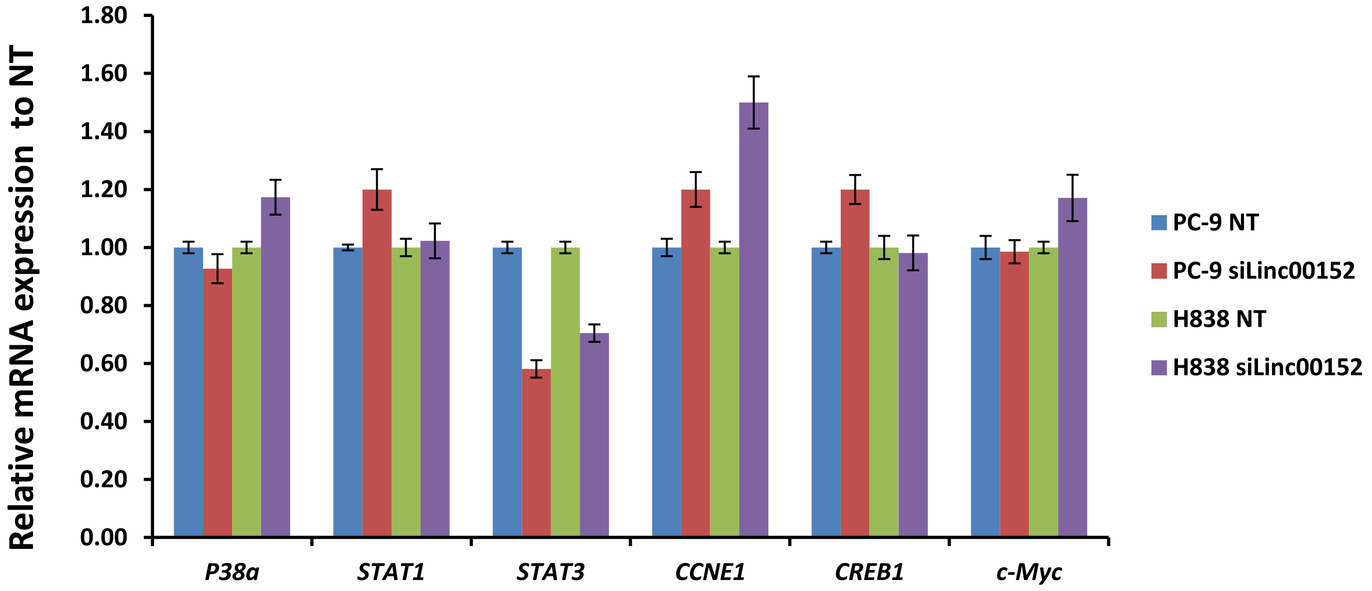


The mRNA expressions of genes in Figure 5B after *LINC00152* siRNA treatment at 48 hours measured by RT-PCR, y-axis is relative mRNA value to NT after adjusted by *GAPDH*. *STAT3* mRNA is decreased by 30-40%, whereas *CCNE1* mRNA in H838 is increased by 1.5 fold. Other genes are not changed.

**Supplementary Figure S6**

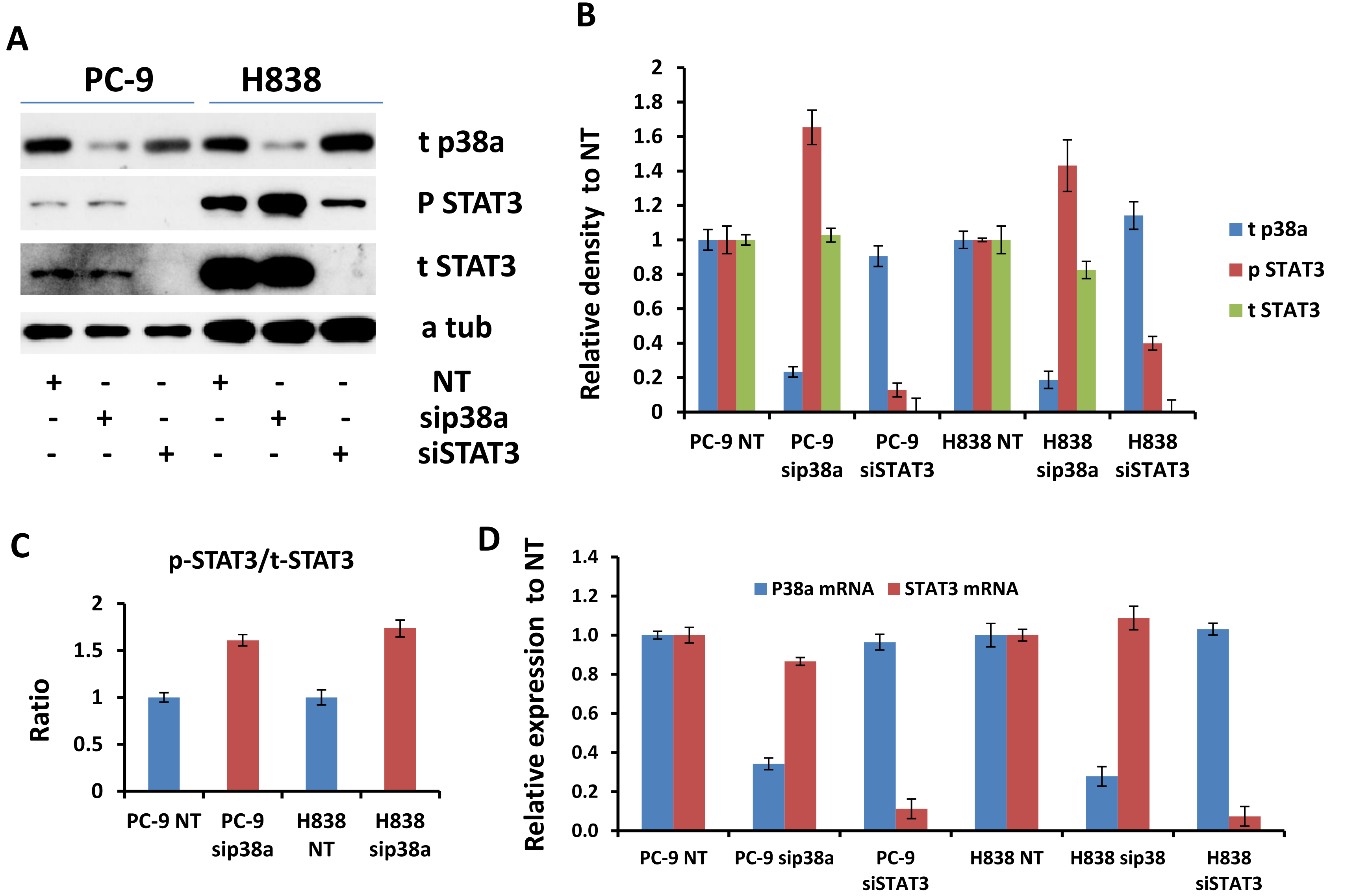


Protein (**A**-**C**) and mRNA (**D**) expression after p38a or STAT3 siRNAs treatment at 48 hours on PC-9 and H838 cell lines. The ratios of p-STAT3/t-STAT3P were increased by 1.6 -1.7 fold (vs. NT) after p38a knockdown (**C**), but the mRNA levels of 38a and STAT3 are not affected each others.
